# Sustaining Healthy Communities in rural settings: lessons from multisectoral teams in Alberta, Canada

**DOI:** 10.1093/heapro/daag055

**Published:** 2026-04-28

**Authors:** Christina Gillies, Courtney Baay, Jacky Ka Kei Liu, Stephanie Patterson

**Affiliations:** Health Intelligence, Evidence & Improvement, Primary Care Alberta, 10030 107 St NW, Edmonton, AB, Canada T5J 3E4; School of Public Health, University of Alberta, 11405 87 Ave NW, Edmonton, AB, Canada T6G 1C9; Health Intelligence, Evidence & Improvement, Primary Care Alberta, 10301 Southport Lane SW, Calgary, AB, Canada T2W 1S7; Health Intelligence, Evidence & Improvement, Primary Care Alberta, 10301 Southport Lane SW, Calgary, AB, Canada T2W 1S7; Faculty of Social Work, University of Calgary, 2500 Universty Drive NW, Calgary, AB, Canada T2N 1N4; Health Intelligence, Evidence & Improvement, Primary Care Alberta, 10301 Southport Lane SW, Calgary, AB, Canada T2W 1S7

**Keywords:** Healthy Communities, rural population, sustainability, health promotion, multisectoral partnerships

## Abstract

The Alberta Healthy Communities Approach (AHCA) supports communities in creating environments that support healthy behaviors and reduce the risk of cancer and chronic disease. In this 4-year project, participating rural communities implemented an iterative five-step process that included establishing multisectoral teams (MSTs) to develop, implement, and evaluate health promotion initiatives aligned with local priorities. Communities received implementation support through mentoring and facilitation, evidence-based tools, and seed funding. This study examined: What factors are essential to sustaining the AHCA in rural communities? Nineteen communities and 258 MST members participated. Community perspectives on factors and strategies influencing sustainability were captured through surveys and focus groups. Data were analyzed using conventional content analysis and codebook thematic analysis, and findings were synthesized narratively. Five key factors were identified, including: strong local leadership, community engagement, municipal support, capacity building, and maintaining collaboration. Strategies that supported sustainability included: developing transition plans and sharing responsibilities across MST members; maintaining ongoing, tailored engagement activities and using multiple communication channels; engaging local government early and aligning initiatives with municipal priorities; identifying training, mentoring, and shared learning opportunities; and investing continuously in relationships and trust building. These findings offer actionable recommendations for strengthening rural health promotion efforts and sustaining MSTs and community-led initiatives. Overall, the study highlights that proactive sustainability planning is a key contributor to the long-term success of community-led health promotion in rural settings.

## Graphical Abstract

**Graphical Abstract:**
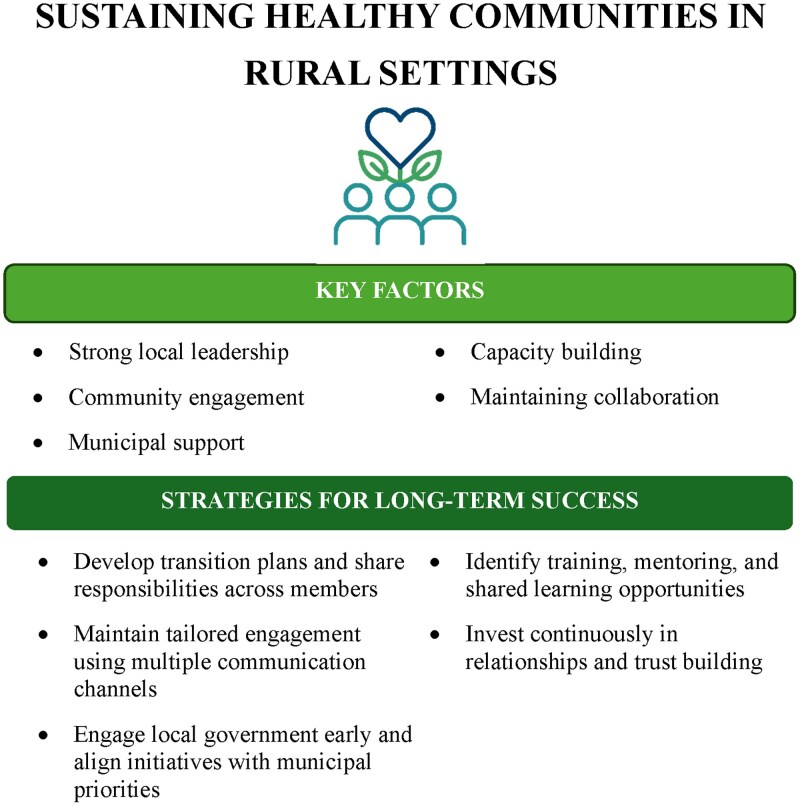

Contribution to Health Promotion

- Strong local leadership and visible municipal support help rural communities initiate, sustain, and grow health promotion initiatives.
- Collaborative relationships grounded in respect, trust, and shared purpose strengthen coordinated community action across sectors.
- Leveraging local strengths, skills, and resources reduces dependency on external funding and supports continuity.
- Proactive sustainability planning, including clear roles and transition processes, increases long-term viability of health promotion initiatives.
- Adaptable sustainability strategies identified in this study can be tailored to diverse rural contexts, enabling communities to develop locally driven, context-specific solutions.

## Introduction

The Healthy Communities Approach (HCA) is a theoretical framework for a settings-based, participatory process by which citizens and partners promote healthy individuals and communities. The approach has been used by cities, municipalities, and communities across the world for decades, becoming or laying the foundation for the standard way in which many organizations promote community health and well-being (Hancock 2009, Hancock *et al*. 2017). In Canada, the Healthy Communities movement has developed in several provinces, including Ontario, British Columbia, and Alberta (Hancock *et al*. 2017, Chaisson *et al*. 2022). The HCA emphasizes a comprehensive, community-driven approach to health and well-being, focusing on factors beyond individual health to improve social, physical, economic, and environmental conditions in ways that are both sustainable and equitable (Hancock *et al*. 2017). Foundational strategies including community involvement, intersectoral partnerships, and asset-based community development are used to develop a wide variety of initiatives aimed at improving overall health and well-being of communities (Hancock 2009).

Community multisectoral teams (MSTs) are partnerships that result when diverse individuals, groups, and parts of society come together to address complex issues affecting a community. A key element of the HCA, these teams involve cooperation and collaboration between all sectors that come together to identify, understand, and generate solutions to local priorities that are relevant to the health and well-being of individuals and communities. Community health promotion initiatives driven by MSTs (hereafter referred to as community-led initiatives) are also a key element of the public health approach to preventing cancer and chronic disease, addressing the social and structural determinants of health, and promoting health equity (Glasgow *et al*. 2023, Honeycutt *et al*. 2023 , Wiggins *et al*. 2025). However, to achieve their potential for long-term community change and improved health outcomes, both MSTs and community-led initiatives must be sustainable over time.

Sustainability can be understood as the ability to maintain community capacity, community-led initiatives, and/or benefits of community-led initiatives in a community over time (Schell *et al*. 2013, Bodkin and Hakimi 2020). Sustainability may be influenced by a multitude of social, economic, political, and environmental factors, including partnerships, funding stability, and organizational capacity (Bodkin and Hakimi 2020, Bacon *et al*. 2022). In maintaining an MST, communities are challenged with preserving communication, relationships, and commitment over time. For instance, interpersonal conflicts due to differences in personal and/or organizational priorities can result in member turnover or MSTs dissolving. Community-led initiatives also need to be supported over time, which requires ongoing investment of human and financial resources, as well as local organizations or groups to agree to lead and integrate the initiatives into their plans and/or budget (Oestman *et al*. 2025). For example, changes to the physical built environment (e.g. paths for walking or biking) require maintenance, cleaning, and repair, as well as ongoing public communication work to promote community-wide usage.

While communities have been taking local actions to improve health and well-being for decades, publicly available data and knowledge concerning the sustainability of the HCA in Canada are limited. Evaluations of the Healthy Cities approach in Europe offer insight into barriers and facilitators for sustainability of this approach (Baum *et al*. 2006, World Health Organization 2022). However, this evidence base may not be transferable to the Canadian context where the HCA differs from the European approach in significant ways (Hancock 2014). In addition, there is a general lack of understanding concerning the factors and strategies influencing sustainability of this approach in smaller municipalities and rural communities across the world. As rural communities face unique barriers, including fewer resources and lack of infrastructure as compared to larger cities, it is important to identify and leverage their strengths to support sustained community health promotion. The purpose of this study was thus to explore the factors and strategies that influence the sustainability of the Alberta AHCA in rural Alberta communities and articulate insights intended to be adaptable across diverse rural contexts to support transferability of lessons learned.

## Materials and methods

### Intervention

The AHCA supports communities to foster healthier environments that enable healthy behaviors to prevent cancer and chronic disease while promoting community health and well-being more broadly. To develop the approach, the HCA was adapted to rural Alberta by the Community Team in Cancer Prevention and Screening Innovation and their collaborating partners. This process occurred in two phases—Phase I (2015–2019) and Phase II (AHCA II) (2019–2023)—with adaptations focused on mobilizing available assets in rural communities and emphasizing initiatives for cancer and chronic disease prevention. The process of developing the AHCA and its implementation strategies and supports has been described in detail elsewhere (Chaisson *et al*. 2022). This paper reports only on AHCA II, which aimed to inform sustainability and scaling.

**Figure 1:**
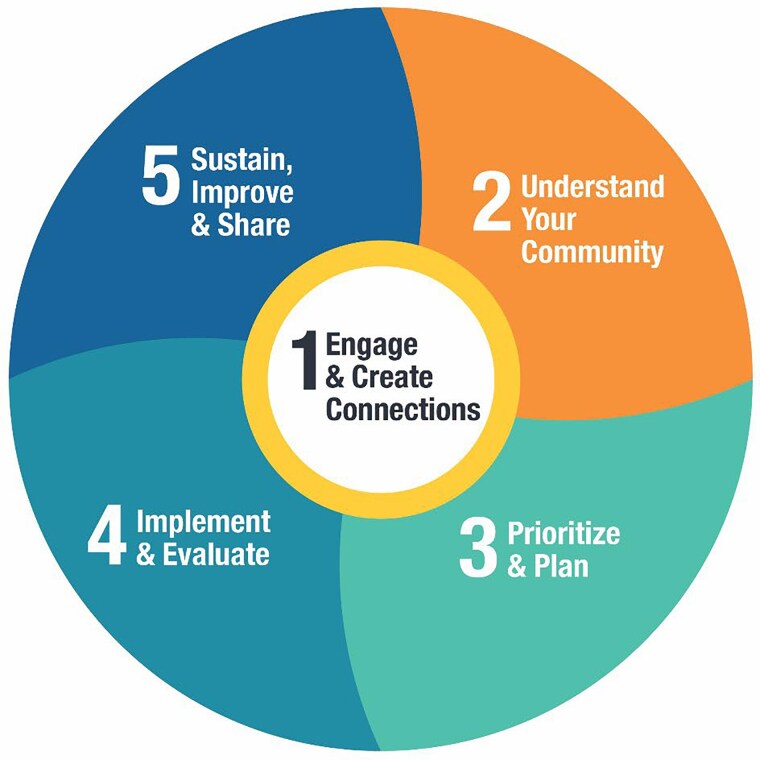
The Alberta Healthy Communities Approach (AHCA) iterative five-step process, which includes (i) engage and create connections, (ii) understand your community, (iii) prioritize and plan, (iv) implement and evaluate, and (v) sustain, improve and share.

Participating communities followed the AHCA process (Fig. 1), which includes five iterative steps. Community MSTs were established and each received implementation support in the form of mentoring from a health promotion facilitator (HPF). Support Partners (SPs) (known locally as leads or co-leads) were individuals from the community who were identified or emerged as leaders during the process. The SPs worked closely with an HPF to champion the process and build capacity within the broader MST. Communities received evidence-based tools and resources, including the Alberta Healthy Communities Action Guide to support implementation; Healthy Places Action Tool to assess their community environments and identify community priorities; Community Capacity Assessment Tool to support collective reflection on community assets, challenges, and readiness for change; and Sustainability Plan to support ongoing action (Chaisson *et al*. 2025, Primary Care Alberta 2026). Communities also engaged in learning and sharing opportunities through a series of Community of Practice (CoP) events and the Healthier Together Communities website, which includes digital and written community stories, evidence-informed strategy kits, community health profiles, and a submission form to share community stories (Primary Care Alberta 2026). Finally, communities received seed funding of $20 000 CAD to support implementation of the process.

### Positionality

The researcher responsible for the qualitative study design is a Canadian-born woman with educational and research training (Postdoc, PhD, MA) in anthropology, community health promotion, public health, and health equity. Her interpretive lens is grounded in social constructivism and pragmatism, and she prioritizes participatory methodologies and qualitative approaches in her work. The research associate involved in data analysis is also a Canadian-born woman with Master’s-level training in sociology, who shares a similar constructivist and pragmatic orientation. Both researchers acknowledge the beliefs, values, and lived experiences they bring to the research process and recognize that these factors shape their engagement with participants, analytical decisions, and data interpretation.

### Study design

This qualitative sub-study explored the question: What factors are essential to sustaining the AHCA in rural communities? The broader study employed a multi-method design, incorporating both qualitative and quantitative data collection through surveys, interviews, and focus groups with SPs, MSTs, and HPFs. This paper specifically reports on qualitative findings that reflect the perspectives of SPs and the wider MST, as these partners are uniquely positioned to provide insights into the sustainability of the AHCA and related community-led initiatives. All participants provided written informed consent and agreed to the use of anonymized data for knowledge translation purposes, including publication.

All knowledge generating activities were conducted in accordance with and assessed using the ethical standards of the “A Project Ethics Community Consensus Initiative (ARECCI)” which provides provincial ethics oversight for quality improvement, evaluation, and health innovation projects. The data underlying this article cannot be shared publicly due to privacy of individuals who participated in the study. Data in support of this research (e.g. analysis files) may be shared on reasonable request to the corresponding author. The study was exempt from Institutional Review Board approval as data collection primarily supported evaluation and quality improvement of the AHCA process developed in Phase I.

### Setting and participants

The AHCA II project involved a total of 19 rural communities in Alberta, which ranged in population size from 522 to 14 436 people. Community MSTs were formed to implement the AHCA and included representatives from different sectors, such as community-at-large, community facilities and organizations, healthcare, schools, and workplaces. MSTs ranged in size from 4 to 30 individuals in the community, with a total of 258 MST members participating in the project.

### Data collection and analysis

#### Support partner surveys

Anonymous online surveys were distributed to SPs by HPFs at three time periods: (i) immediately following completion of the AHCA II project in their respective community, (ii) 6 months post-completion, and (iii) 1-year post-completion (three surveys total). The first survey was sent in May 2022 and the final survey in December 2024. The initial survey included open-ended questions (*n* = 7) designed to identify factors that enabled implementation and maintenance of community-led initiatives. The second and third surveys included the same open-ended questions (*n* = 4), along with dichotomous items (*n* = 4) (e.g. “Has your community maintained healthy community initiatives that were developed as part of AHCA?”) and associated bridging questions (e.g. “If yes, please describe the factors that have enabled the initiative(s) to be maintained over time; If no, please describe the factors that lead to the initiative(s) not being maintained over time”). These surveys were intended to capture data related to sustainability strategies and the collective impact of the AHCA.

SPs received survey links via email and were given two weeks to respond. One follow-up reminder email was sent. Surveys were completed by up to 15 SPs representing 12 communities (SP Survey 1: *n* = 15; SP Survey 2: *n* = 15; SP Survey 3: *n* = 8), noting that more communities had more than one SP. Survey data were analyzed by one research associate (CB) using conventional content analysis (Hsieh and Shannon 2005). A second researcher (CG) supervised the analysis process and served as a peer-debriefer.

#### Focus groups

Between May 2023 and January 2024, 18 community MSTs participated in end-point focus groups. One MST had dissolved prior to data collection due to interpersonal conflict and turnover and therefore did not participate. Focus groups were conducted via Microsoft Teams by a male, Master’s-trained evaluation associate (J.K.K.L.) using a semi-structured interview guide. Participants did not have a prior relationship with the interviewer but were introduced by their HPF. Focus groups lasted approximately one hour and ranged from 2 to 10 participants, with a total of 86 participants. One scheduled focus group was attended by only one participant and proceeded as an interview using the same interview guide as the focus groups.

Discussion topics included experiences with AHCA implementation, sustainability planning, and perceived community impacts. All transcripts were verified for accuracy and anonymized. Transcript analysis was conducted by one research associate (C.B.) using a “codebook” thematic analysis (TA) approach (Braun and Clarke 2021a) and NVivo 12 software. Unlike “coding reliability” TA—which emphasizes agreement among multiple coders—a codebook TA approach does not use consensus coding or inter-rater reliability as markers or measurement of quality (Braun and Clarke 2021b). Nevertheless, a second researcher (C.G.) supervised the analysis process and supported data interpretation and synthesis.

#### Integration and reporting

Themes resulting from the focus groups were integrated with survey findings and are reported below through a narrative synthesis.

## Results

Several factors and strategies were identified as influencing the sustainability of MSTs and community-led initiatives. Table 1 provides the associated factors identified by participants as instrumental to sustainability alongside illustrative quotes from surveys (identified using ‘S1’, ‘S2’, and ‘S3’) and final focus groups (identified using ‘FG’). Each factor and their complementary strategies are described in detail below.

**Table 1:** Factors influencing sustainability of rural community action.

Factor	Description	Illustrative quote(s)
Strong local leadership	An individual (or small group) who acts as a lead or “champion” at the local level. A local lead possesses the skills, knowledge, and determination to advance the process alongside a community team.	“A strong lead is able to respectfully facilitate the ability for everyone at the table to have a voice thereby promoting inclusion and participation within our team.” (P15, S1)
Community engagement	Actively engaging members of the MST and broader community over time to support action planning and the overall success of healthy community-led initiatives. This includes engaging active participation from community members through volunteerism, sharing resources, and promoting, and attending events.	“Community engagement and the connection is key. So having that solid group of community associated community members that are passionate about seeing progress, and seeing success, and seeing their community thrive is important.” (P3, FG)
Municipal support	Support, encouragement, and formal endorsement from locally elected or appointed officials and other decision-makers.	“When you’ve got that municipal buy in, stamp of approval, and endorsement, it’s so much easier to do the work that you need to do.” (P46, FG)
Capacity building	Strengthening the local knowledge, skills, and resources required to effectively implement the process and address barriers and challenges over time.	“We have been able to leverage various types of funds to continue to grow our ability to enhance community wellness. It has also led to being connected with a wide array of resources that support our goals. We have been able to broaden our membership and partner with other groups in the community who share our mandate.” (P15, S2)
Maintaining collaboration	Embedding collaboration among diverse partners as a core value and committing to long-term connections within and outside the community built on shared vision, trust, and communication.	“Gathering ideas and continuing the messaging might bring more people to the table eventually. And then just keeping those connections in the community as a nucleus and branching out and reaching out to other connections in the community.” (P36, FG)

### Strong local leadership

Participants emphasized that the sustainability of MSTs and community-led initiatives was strengthened by the presence and guidance of at least one dedicated community lead. Participants identified that it is essential for the individual(s) in this role to have a passion for improving community health and wellness, as well as the skills, knowledge, and determination to initiate and/or maintain momentum of the AHCA process. Established relationships and connections within the community, combined with strong interpersonal, communication, organizational, and relationship-building skills, were viewed as critical to success in this role.

However, not all experiences reflected this ideal. One participant shared that they felt they were not the right fit as a lead, explaining:

I shouldn’t have been the lead. I filled the gap when there was no community member willing to step forward to lead the group. I should have waited and kept looking for a true community member to lead the group […] it led to the community thinking [I was] going to do all the work for them instead of with them. (P11, S1)

Another participant explained: “Going forward, anybody else that takes on this initiative, it would be highly suggested that person be able to be immersed in some of those community engagement opportunities” (P55, FG). These reflections underscore the importance of recruiting a local leader who is present, engaged, and committed to fostering shared ownership and supporting long-term success.

While most participants indicated that they planned to continue their leadership roles within the community, some had developed an internal transition plan and/or intentionally trained others on an ongoing basis to ensure that initiatives would be supported long-term. As one participant explained, “If we’re training the people around us to [continue AHCA] and be leaders and not just followers and attendees [that] will help us with sustainability” (P28, FG). Sharing responsibilities and intentionally growing the MST over time was also recognized as supporting sustainability. For example, one participant stated that they were “hoping to continue to encourage others to take on shared responsibility within our team, as well as continuing to recruit members who have a passion for improving well-being in our community in order to sustain or grow on initiatives we have already started or completed” (P15, S1).

Conversely, the lack of a dedicated team was found to hinder sustainability of the AHCA process. One MST “disbanded and had no one else available to take over” once dedicated support from an HPF ended, and the community was also unable to sustain their initiatives as there was “no one dedicated to carrying on the workload at the local level” (P10, S2). Related to this finding, participants emphasized that it was necessary to protect team leads from stress caused by overreliance, responsibility, and burdensome commitments by planning for the level of investment and extent of support required to implement the AHCA. As one participant stated, “Volunteers can become burnt out. Down times are required to allow for people to recharge their energy supply” (P4, S1). Another participant shared similar concerns in mentioning that the time commitments for community-led initiatives may lead to some members “taking on more [than they are] able to do” (P2, S2). As such, it is important to ensure that collaboration activities and community-led initiatives are manageable, acceptable, and realistic for all MST members to reduce turnover and support sustainability.

### Community engagement

Participants also identified that actively engaging the involvement, participation, and support from community was essential for informing action planning as well as supporting the momentum and overall sustainability of the AHCA. Most focus group participants recognized that it would have been difficult to effectively implement and sustain community-led initiatives without community engagement and support. As one participant reflected: “A big thing that I would say going forward is having that plan [to] continuously keep people engaged in the community, engaged in the initiative, and really understanding what they're doing and why they're doing it, having additional opportunities for people to join if they wanted to join because it is quite a lengthy process” (P60, FG). Another participant reflected on the importance of continuing to build relationships with the broader community over time, explaining “ [It’s] really about building community […]we just need to always be aware that we have to keep paying attention to this. We can't sit back and think it's done […] we need to make sure that even if we don't have grants that this is just such a part of the fabric of the community” (P4, FG).

Accordingly, tailored engagement strategies were used by MSTs to build and maintain connections and partnerships within their communities. For instance, involving residents in active planning sessions and promoting initiatives through media and word-of-mouth communication were strategies used to build connections, strengthen partnerships, and gain support from diverse community members. When asked how community-led initiatives had been enhanced, one participant mentioned that “The group continues to actively involve the community in upcoming planning sessions to ensure the programs the group continues to run what is needed in our community” (P14, S2). Another participant shared that their “community has continued on with building community connection by hosting different events/workshops promoting wellness” (P13, S2), while another stated that a community-led initiative had “grown exponentially with more community support, allowing [the MST] to create a wider reach in the community, and encourage even greater participation” (SP 15, S2).

Participants acknowledged that community-led initiatives rely on the hard work and dedication of MST members and the broader community alike to operate efficiently and sustainably. As one participant shared: “the voice of the community is heard through our team, which is important when making decisions that affect community. They are a support, sounding board, and have access to people and resources to move initiatives forward” (P5, S3). The participant further explained that it is important to have “People in place to do the work, and initiatives that remain relevant to our community” (P5, S3). Conversely, several participants reflected that difficulty in finding and/or retaining community volunteers and resources hindered sustainability of the AHCA.

For example, one initiative depended on families to plant and harvest community gardens over the seasons and another relied on donations to support their Lending Library (which allows for the borrowing of health-related resources, goods, and services). One participant shared their perspective that in smaller communities “there’s often members of a community that sit on every single board, always volunteer their time, and it's always the same people. So, I guess I always worry about that about them burning out and needing to recruit more people to step up and help carry on all the work that we're doing” (P5, FG). In recognition of the importance of community engagement and participation, consideration of potential volunteer burnout and striking a balance between involving and asking too much of community members were identified as important to the maintenance of community-led initiatives.

### Municipal support

Another factor identified by participants was the need for support, encouragement, and formal endorsement from local government entities that organize and manage public services as well as create and enforce local laws, bylaws, and policies. Many participants recognized that political support and commitment from elected (or appointed) officials in rural municipalities provided the necessary leverage to implement and sustain the AHCA in their communities. As one participant noted, “the involvement from local levels of government—in our case, the municipality and the county—was critical” (P23, FG). Another participant identified “a newly formed wellness committee [that] has been created with municipal council commitment” (P2, S2) enabled the development of new community-led initiatives. In some cases, the municipality also provided funding to support community-led initiatives: “Financial support from the municipality […] has been important” (P5, S3). Such reflections indicate that support from local officials and alignment with municipal priorities enhance the strong collaborations and partnerships required to sustain the AHCA process.

Indeed, participants found that engaging partners from the municipal government at the very outset of the AHCA process was a crucial strategy to secure long-term political support for the implementation and maintenance of community-led initiatives. As one participant reflected, “I believe that groups like this have to have town and county representation […] I think if you don't have any buy in from the town and county, it would make things a lot, that make things a lot more challenging” (P32, FG). The importance of this factor was further emphasized by a community that had struggled to engage their municipalities and identified this lack of support as a barrier to sustainability: “It has been considerably challenging to engage our municipalities in championing the work we are doing. The lack of collaboration from them has definitely been a barrier to seeing success in some of earlier identified action plans” (P15, S2).

### Capacity building

Communities shared that leveraging and strengthening existing individual and collective knowledge, skills, and resources was a necessary factor for effective implementation and proactively addressing challenges over time. By building capacity upon existing assets, most communities were enabled to lead action independently without dependency on external support or single-source funding. To support this sustainability factor, MST members identified and accessed evidence-based supports and resources to provide ongoing training, support, and inspiration to maintain initiatives over time. Participants found HPF mentorship, assessment tools, and learning from other communities to be especially useful resources to support AHCA sustainability. Community awareness of, access to, and familiarity with using the resources strengthened the overall self-sufficiency of communities to maintain their initiatives.

Building capacity within the community and MSTs was also a strategy used to aid communities in securing funding to sustain and expand community-led initiatives developed and implemented through the AHCA. Human and financial resources were frequently identified as both barriers and facilitators to sustainability. As one participant explained, “Everybody has great ideas […] It is just finding funds and that's definitely a challenge” (P8, FG), while another shared, “there are some really imaginative people in our community that have great ideas, but not always the resources to implement the ideas” (P2, S1). Given these reflections, it is encouraging that the AHCA was reported to effectively equip communities with the necessary skills to secure additional funding and attract volunteer support, which they used to enhance existing programs and develop new initiatives. For instance, one participant shared that the AHCA process had “allowed us to apply with success for our second year of grant funding through the rural mental health initiatives, which has, in turn, expanded our ability to target wellness and engagement across the lifespan” (P15, S2).

Furthermore, MSTs found learning from the experiences of other communities that had previously implemented AHCA to be particularly useful as these learnings offered insights into effectively maintaining initiatives over time and inspiration for future community-led action. For instance, one participant noted that provincial meetings provided the opportunity to “[listen] to ideas of what other communities were doing, where they accessed their materials from, what went well/didn't go so well” (P2, S1). The participant further acknowledged that “It’s much better to share ideas and how to implement them than to reinvent the wheel over and over” (P2, S1). Overall, community members found that the strengths-based approach utilized in the AHCA that focuses on mobilizing existing resources, skills, and knowledge as well as learning from experience supported long-term sustainability of AHCA.

### Maintaining collaboration

Lastly, participants identified that maintaining collaboration among MST members and partners—while continuing to establish new connections—was essential in sustaining the AHCA. One participant mentioned that their community had maintained their MST due to the “strong partnerships that had been established and coalition members [being] committed to health and wellness” (P6, S2). Indeed, utilizing the distinct skills, knowledge, connections, and resources of community members was viewed as key to sustainability. As one participant explained:

We figured out along the way if we want things to work here in a small community that's rural […] that we had to work together. We need to pull resources, both financially, manpower, ideas […] I think we've just learned a long time ago that we are much better together than we are as individual agencies fighting for resources. We get more out of it if we can collaborate. (P28, FG)

These reflections underscore that collaboration is not just beneficial but essential for rural MSTs to thrive. By pooling resources and working collectively, communities can address the resource and capacity challenges that often hinder progress in smaller, rural settings. This sentiment was echoed by other participants, who emphasized that “when everyone works together and uses their skillset and knowledge of their community, projects come together very easily” (P8, S1), and that “working with each other instead of in silos is a much better way to make a difference” (P14, S1).

To support this factor, participants recognized the importance of ongoing investment into relationships and trust building among community partners. Participants identified that it was crucial that MSTs include diverse individuals in terms of background, expertise, and lived experiences to bring unique knowledge, perspectives, and relationships to the table. As one participant explained, “Good relationships with those in the community is important. Having a wide variety of people at the table means you have connection to the community as a whole and access to a wider variety of assets” (P12, S1). Maintaining regular meetings and providing opportunities for MSTs and community partners to connect was recognized as another strategy to support sustainability. One participant said that their MST members “Continue to meet in person or over zoom monthly and I believe it helps to maintain consistency” (P2, S3). However, the importance of considering the convenience and time commitment of participating in the AHCA for team members was also stressed. Examples provided to mitigate challenges included providing virtual meeting options and adjusting meeting frequency as required.

Finally, participants recognized that it was essential that MSTs anticipate and address issues such as interpersonal conflict and staff turnover to sustain strong, resilient partnerships and collaborations over time. To this end, some participants noted that their MSTs had developed sustainability plans that encouraged open communication about team dynamics, responsibilities, and expectations. Participants also shared that nurturing strong collaborations would inherently ensure that the MST and their initiatives were well-positioned to be transferred to and upheld by other groups and organizations in the community as needed. As one participant explained, “our Community Wellness Team has been able to continue through the interagency group that was started as an initiative […] many of the team members are part of this interagency group, which has taken on the name, vision, mission, and terms of reference from the original wellness team” (P3, S2).

## Discussion

This study explored the factors and strategies identified by members of rural MSTs in Alberta, Canada as contributing to the sustainability of an adapted HCA. Findings from our study strongly align with international evidence on sustaining complex, community-driven health promotion initiatives (Shelton *et al*. 2018, Bodkin and Hakimi 2020, Glasgow *et al*. 2023, Oestman *et al*. 2025, Wiggins *et al*. 2025). For example, a 3-year follow-up of a Danish community-based health promotion initiative demonstrated that while coordinated actions diminished after project completion, its underlying values and principles—including integration, participation, and context awareness—continued to influence partners’ mindsets and municipal policy (Pedersen *et al*. 2023). This suggests that sustainability may be achieved not only through maintaining specific activities but by embedding values and ways of working into local systems and governance structures. Furthermore, partners identified the absence of a dedicated coordinator and funding as major barriers to sustaining collective action, reinforcing the importance of early planning for structural integration and resource continuity.

While sustainability factors identified in international studies may apply broadly to community-led health promotion initiatives, not all are directly transferable to implementing the HCA in Canadian rural context. As such, the sustainability factors and strategies identified in this study offer evidence for other rural communities and smaller cities, villages, and towns in rural settings in Canada to consider and revisit over time to support the maintenance of both community-led initiatives and the MST itself. Moreover, the sustainability factors and strategies identified here are conceptually consistent with global health promotion principles (Nutbeam and Muscat 2021) and are adaptable to rural settings internationally, including those with limited resources or decentralized governance.

Our study reinforces the fundamental importance of community engagement in sustaining health promotion initiatives at the local level. Community engagement refers to genuine involvement and active participation of individuals, groups, and organizations within a community to make decision, generate solutions, and take action to achieve change (Dooris and Heritage 2013, Gilmore *et al*. 2020). The approach exists along a continuum, with levels of involvement ranging from passive participation to full leadership (Hancock 2009). The AHCA facilitates the highest level of involvement by fostering partnerships between diverse organizations—such as local government officials, health departments, community health organizations, schools, and residents—which collaborate on initiatives to promote and improve health in the community. This supports sustainability by reflecting the knowledge, priorities, and perspectives of the broad community; instilling feelings of ownership, commitment, and responsibility; increasing local acceptance and commitment; and encouraging collaboration across social, economic, and political lines to enhance alignment and avoid potential conflict (Bodkin and Hakimi 2020, Barasche-Berdah *et al*. 2022).

Complementing existing literature, this study highlights the importance of community engagement to not only inform initiatives but also maintain them through participation, volunteerism, and shared resources. This finding may reflect the limitations that rural and remote communities in Canada face in capacity due to factors such as infrastructure and service gaps, limited access to opportunities for training and education, geographic isolation, and low population density. Given these issues, rural communities often depend on intentional partnerships and collaboration across sectors to leverage existing assets (Chaisson *et al*. 2022). To support the sustainability of the AHCA, it is thus important for rural MSTs to allocate resources to ongoing community engagement (Glasgow *et al*. 2023) as well as attract and retain volunteers by understanding their motivations and addressing barriers to participation (Stukas *et al*. 2016). An effective communication strategy for both MST collaborations and community-led initiatives that emphasizes locally relevant messaging and operates at multiple levels can further bolster awareness and participation among residents to support sustainability (Love *et al*. 2024).

This study also highlighted the particular importance of engaging local leads and elected (or appointed) officials to sustain the AHCA. It is crucial to identify and engage a lead who is an active member of the community and possesses the skills, enthusiasm, and determination to move the AHCA process and way of working forward. Local leadership and structure sustain community health promotion efforts as they provide the consistency and advocacy needed to strengthen and maintain community action (Bodkin and Hakimi 2020). As such, individuals must be secured to champion the AHCA prior to implementation, while sharing responsibilities with a wider group throughout the process (Bodkin and Hakimi 2020, Morgan *et al*. 2022). To reduce burden and protect against staff burnout and turnover, it has been suggested that MSTs adopt a decentralized, collaborative leadership model whereby leaders emphasize open communication, distribution and delegation of tasks, and collective decision-making (Wiggins *et al*. 2025). Use of this model in addition to having a clear succession plan for MST members will help to mitigate negative effects resulting from changes in team membership and leadership.

Support, encouragement, and endorsement from local government can further strengthen and sustain rural community action. The commitment of elected and appointed officials and other decision-makers within rural communities can enable sustainability through direct political action. For instance, municipal leaders hold the power to develop, enact, and enforce legislation, regulations, and policies that reinforce healthy community-led initiatives (Bodkin and Hakimi 2020, Nutbeam and Muscat 2021). Political leaders can also convene intersectoral partnerships; mobilize resources to support community-led initiatives; and communicate public support to generate interest, participation, and acceptance among citizens (Schell *et al*. 2013). As other studies have found, municipal involvement can both encourage and hinder sustainability of community action as the political environment shifts over time (Bodkin and Hakimi 2020). It is thus critical that rural communities engage with local government on an ongoing basis by sharing information about their activities and developing initiatives that are integrated with municipal plans, strategies, and policies.

The sustainability of the AHCA is also influenced by opportunities to leverage and build existing capacity within the community. In the context of this study, “capacity building” is understood as the development of knowledge, skills, and systems to enable effective actions to improve health (Nutbeam and Muscat 2021). Both partnership and capacity building establish a “multisector ecosystem” for health that is critical for the effective implementation and maintenance of community-led initiatives (Raber *et al*. 2025). As a lack of human and financial resources has been recognized as common barrier to maintenance of community-led initiatives (Bodkin and Hakimi 2020, Barasche-Berdah *et al*. 2022, Wiggins *et al*. 2025), it is particularly important for rural communities to establish partnership networks to facilitate quick and positive responses to requests for support (Raber *et al*. 2025). Such networks can establish a shared agenda or priorities, pool human and material resources amongst diverse groups and organizations, and engage in shared learning and skill development to support sustainability (Bodkin and Hakimi 2020, Wiggins *et al*. 2025). Sustainability activities could include preparing joint funding proposals and regularly connecting with other communities to share best practices and lessons learned to inform planning and improvement.

Perhaps the most challenging strategy identified by MST members participating in the AHCA is the need to maintain and nurture interpersonal connections and collaboration among diverse partners over time. Shifts in political, social, cultural, and economic context within and outside the community can compromise sustainability by increasing staff turnover, triggering interpersonal conflicts, and reducing trust (Barasche-Berdah *et al*. 2022). As rural communities may have fewer opportunities to gain the skills, talents, and experiences for health promotion (i.e. human capital) found in urban communities, intentional investment into sustainable collaboration is a necessity. It is thus important that teams have regular connection points (i.e. virtual and in-person meetings) and establish collaborative problem-solving and conflict management processes to maintain trust and open communication (Rice and Hancock 2016, Wiggins *et al*. 2025). Establishing a shared vision, values, and goals to commit to continually working toward as a collective group—while remaining adaptable to changing context—can further support sustainable collaborations (Oestman *et al*. 2025, Wiggins *et al*. 2025).

In addition to considering and implementing the strategies identified in this study, MSTs can actively evaluate sustainability over time to maintain collective action. A number of tools and frameworks have been developed to assist public health professionals and communities in defining, achieving, and evaluating sustainability (Center for Disease Prevention and Health Promotion 2013, Bodkin and Hakimi 2020). For instance, the Program Sustainability Assessment Tool has been developed and validated to measure sustainability capacity of community-level partnerships and health initiatives (Hall *et al*. 2021, Bacon *et al*. 2022).

A website that includes tools and resources specific to the AHCA has also been developed, which includes the Alberta Healthy Communities Action Guide and Community Capacity Assessment Tool to provide guidance on sustainability (Chaisson *et al*. 2025, Primary Care Alberta 2026). The use of such tools will assist MSTs in reflecting upon and improving their processes as well as demonstrating the impact of their community-led initiatives. As the AHCA will now be operationalized as the “Healthier Together Communities Approach,” the Healthier Together Communities website will continue to support scaling and sustainability of the approach across the province. Finally, strategic knowledge sharing with partners and decision-makers further supports sustainability by displaying the value of the AHCA to generate enthusiasm, increase partner buy-in, and attract additional funding support (Wiggins *et al*. 2025).

### Strengths and limitations

This study was conducted over three time periods with members of communities responsible for implementing the AHCA, which provides rich, experienced-based evidence, and reflective insights. However, not all communities were represented in the data as one MST had dissolved by project end and SPs from six communities did not participate in all the voluntary follow-up surveys. One focus group became an interview which enabled deeper reflections that were not influenced by group dynamics. The range in focus group participants (2–10) may have also affected findings as smaller groups (2–3) may have captured less diversity of viewpoints and larger groups (7–10) may have reduced the depth of individual insights. As such, this study has limitations in capturing all factors influencing sustainability as well as providing context concerning why the AHCA may not have continued past project end in some communities.

As the longest follow-up was 1-year post-implementation, it is possible that additional enablers and barriers to sustainability may have arisen in participating communities. Further research and evaluation are required to determine if and to what extent HCAs are sustained long-term in diverse communities in Canada. As generalizability may be constrained by the Canadian rural context, future work should also examine applicability of sustainability strategies identified in this study in other rural regions internationally to strengthen cross-context transferability.

## Conclusion

This study identifies key factors that rural communities and the organizations supporting them should consider when implementing the AHCA or similar community-led health promotion approaches. Although sustaining community action is emphasized in the last step of the AHCA, the iterative nature of the process underscores the importance of integrating sustainability planning from the outset. The factors highlighted in this study offer communities practical guidance for what to plan for, monitor, and evaluate over time to strengthen the longevity of their collaborations and initiatives. The strategic recommendations generated through this research can further assist communities and organizations that collaborate with them in fostering conditions that enable long-term sustainability. By embedding sustainability planning early and intentionally, rural communities can enhance their capacity to implement enduring health promotion initiatives that contribute to improved health and well-being for current and future generations.

## Data Availability

The data underlying this article cannot be shared publicly due to privacy of individuals that participated in the study. Data in support of this research may be shared on reasonable request to the corresponding author.
